# A modified method for preparing meiotic chromosomes based on digesting pollen mother cells in suspension

**DOI:** 10.1186/s13039-015-0184-x

**Published:** 2015-10-24

**Authors:** Jiangbo Dang, Qian Zhao, Xing Yang, Zhi Chen, Suqiong Xiang, Guolu Liang

**Affiliations:** Southwest University, College of Horticulture and Landscape, No. 2 Tiansheng Road, Beibei District, Chongqing, 400715 China

**Keywords:** Meiosis, Pollen mother cells, PMC wall, Suspension, Protoplast

## Abstract

**Background:**

Meiotic chromosome preparation is a key step in plant meiotic research. Pollen mother cell (PMC) wall elimination is beneficial to cytogenetic experimental procedures. Without wall interference, these procedures are easier and more successful. In existing methods it is difficult to eliminate PMC walls completely and uniformly. In this paper, we present an improved method for digesting PMC walls, and one for providing massive chromosomal spreads on a slide for other cytogenetic experimental procedures.

**Results:**

Three plants were selected to exhibit the modified meiotic chromosome preparation method. PMCs were dispersed as single cells and incubated in a mixed enzyme solution (3 % cellulose + 0.3 % pectinase + 1 % snailase) for 1.5–2.5 h. In total, 28.28 % cells were lost during this process. There were 800–1900 spreads on every slide and no PMC wall interference was found on any of the slides. The spreads were also evenly distributed on the slides. More spreads were obtained when PMC and protoplast densities in the suspension were increased. All three plants’ spreads were successfully used to locate a 5 s rDNA conserved sequence. The *Nicotiana* hybrid’s spreads were successfully used to identify the hybrid’s parental genome.

**Conclusion:**

This is an alternative method for meiotic chromosome preparation. Through this method, PMC walls can be completely and uniformly eliminated, and hundreds of spreads on every slide can be obtained. These spreads can be successfully used for DNA *in situ* hybridization.

## Background

Meiotic chromosome preparation plays a major role in meiotic research, and many methods have been employed [1, 2]. These methods can be primarily divided into two types. In one type, pollen mother cells (PMCs) are spread without wall elimination. In the other, PMCs are spread after PMC wall elimination.

Originally, PMCs were squeezed on slides and immediately squashed by cover slips. Chromosomes were released by tapping or pressing [3, 4]. The squash-based methods are simple and fast, and many excellent spreads have been obtained using this method. Thus, it has been widely used in plant cytogenetic research [5–7]. Squash-based methods are mainly employed for karyotype analyses, and are used less for *in situ* hybridization (ISH) [8]. Later, new methods without PMC wall elimination were introduced, but their use was limited [9–12]. Interference from PMC walls and wall fragments limits the use of these methods.

Methods with wall elimination were developed later [13–16]. The methods described by Zhong *et al*. [13] and Ross *et al*. [16] were successfully used in spread preparation for fluorescence *in situ* hybridization (FISH). These methods and some modifications have been widely used in the molecular cytogenetic analysis of meiosis in rice (*Oryza*), *Brassica*, potato (*Solanum tuberosum*), cotton (*Gossypium*), and lily (*Lilium*) [2, 17–21]. In these methods, whole anthers/buds are incubated in mixed enzyme solutions. Unfortunately, anther and/or bud walls were not permeated by the enzyme solution and PMCs grouped together. Thus, PMC walls could not be totally eliminated using low concentrations of enzymes and short digestion times [18]. Although higher concentration enzymes and longer digestion times may eliminate the walls completely, many PMC protoplasts are also degraded. Thus, PMC walls cannot be digested uniformly by incubating the whole anther in a mixed enzyme solution.

In this paper, we improved a method to digest PMC walls completely and uniformly. The key step is the dispersion of PMCs as single cells incubated in a mixed enzyme solution. Prepared spreads were used in FISH and genomic ISH (GISH). To present this method, three different plants were used as materials. We hope that the method provides a new tool for plant meiotic chromosome preparations in cytogenetic research.

## Results

### Loss ratio of cells

A *Nicotiana* hybrid was used to test the cell loss ratio during chromosome preparation using this modified method. There were 115.36 ± 14.65 PMCs per μl suspension when PMCs were squeezed from anthers into 200 μl distilled water at step 2. There were 1,654.67 ± 81.73 meiotic spreads counted, which calculated to a 28.28 % cell loss.

### Meiotic spreads prepared by this modified method

**Fig. 1:**
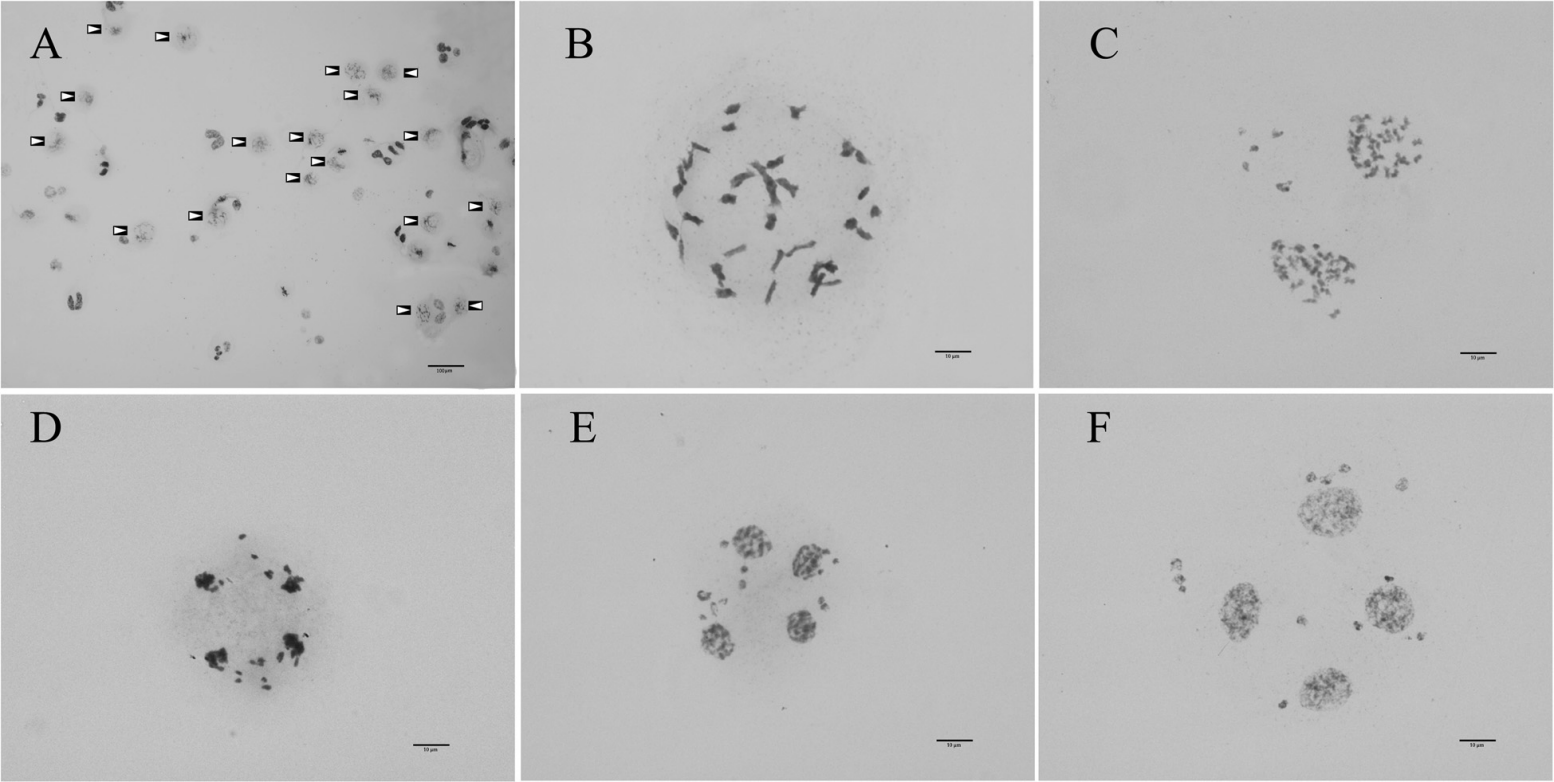
*Nicotiana* hybrid meiotic spreads prepared using the modified method. **a** Meiotic spreads at 100× (arrows); **b** Diakinesis/metaphase; **c** Telophase I; **d** Anaphase II; **e** Telophase II; **f** Tetrad stage

Approximately 800–1900 meiotic spreads were obtained on every slide. There were no spreads containing walls or wall fragments, and no wall fragments were found on the slides (Fig. 1a). Thus, all of the PMC walls were completely and uniformly eliminated in the mixed enzyme solution after a 1.5–2.5 h incubation.

More spreads were obtained when the PMC density in suspension was increased at the incubation step. We obtained the same results when the protoplast density was increased at the resuspension step.

**Fig. 2:**
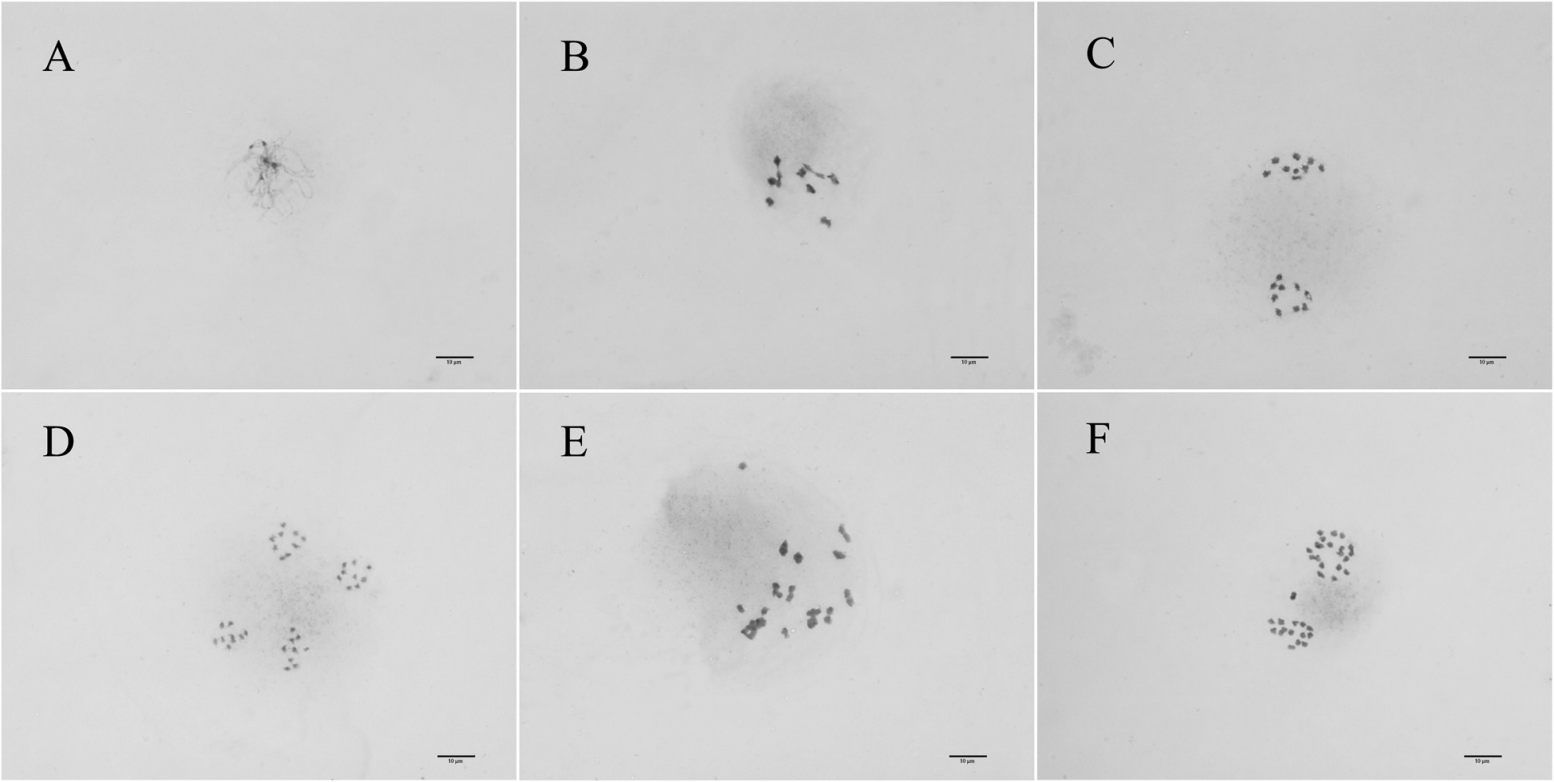
*Cabbage * meiotic spreads prepared using the modified method. **a** Diplotene stage of a diploid plant; **b** Metaphase (nine bivalents) of a diploid plant; **c** Telophase I of a diploid plant; **d** Telophase II of a diploid plant; **e** Metaphase of a tetraploid plant; **f** Telophase I of a tetraploid plant

**Fig. 3:**
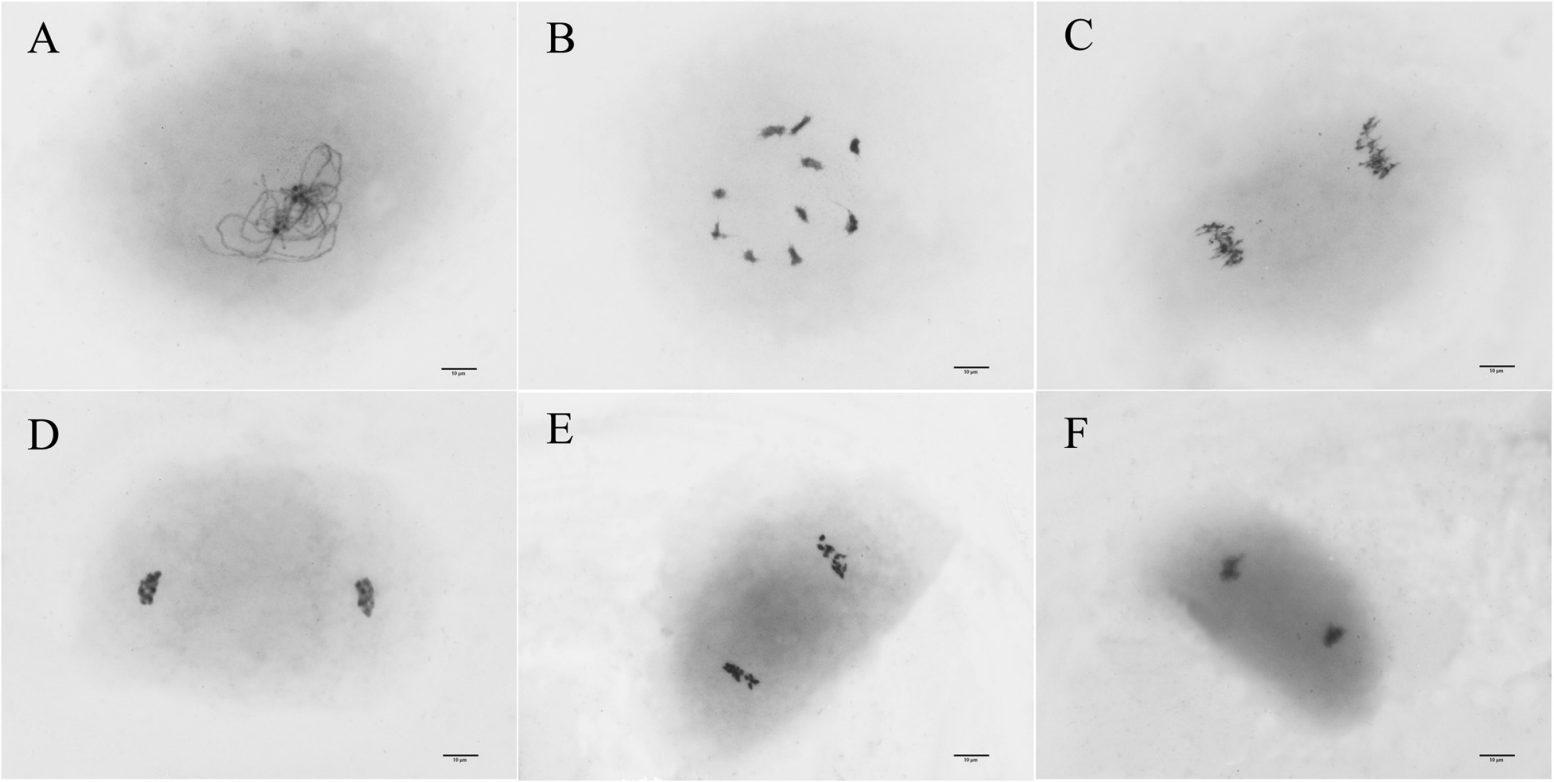
Maize meiotic spreads prepared using the modified method. **a** Diplotene stage; **b** Metaphase (10 bivalents); **c** Anaphase I; **d** Telophase I; **e**, **f** Daughter cells at telophase II

Many nice spreads suited to karyotype analyses were obtained. The *Nicotiana* hybrid’s meiotic chromosomes at different stages during meiosis are clearly shown in Fig. 1 (b–f). Distinguishable spreads of diploid and tetraploid cabbage (*Brassica oleracea* L.) at different stages are shown in Fig. 2. Maize (*Zea mays* L.) meiotic chromosomes before the second meiotic division are shown in Fig. 3.

Interference from the cytoplasm in maize spreads was more obvious than that in the other two plants. This may be because the cytoplasm in maize PMCs was originally thick. However, the thick cytoplasm did not affect chromosome recognition. Unfortunately, complete spreads after telophase I were not found on the slides (Fig. 3). This is because PMCs divide into two separate daughter cells within the wall after telophase I, but the two daughter cells move too far away from each other after the wall is digested.

### 5 s rDNA conserved sequence location on meiotic chromosomes by FISH

**Fig. 4:**
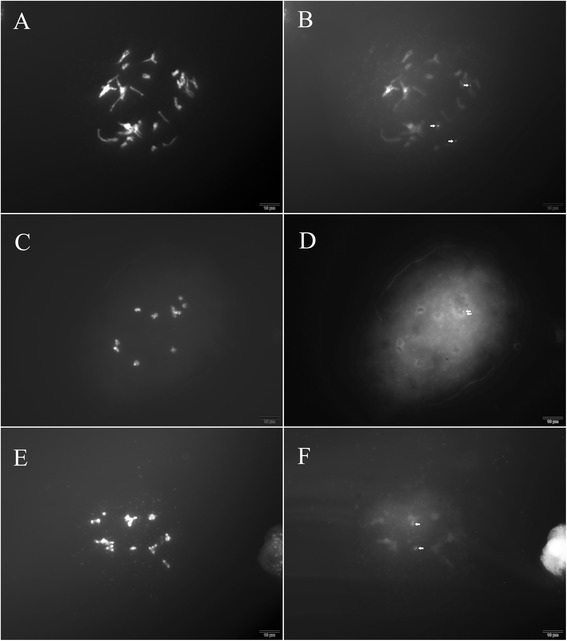
Location of the 5 s rDNA conserved sequence in metaphase chromosomes during meiosis. **a**, **b** *Nicotiana* hybrid; **c**, **d** Maize; **e**, **f** Tetraploid cabbage

**Fig. 5:**
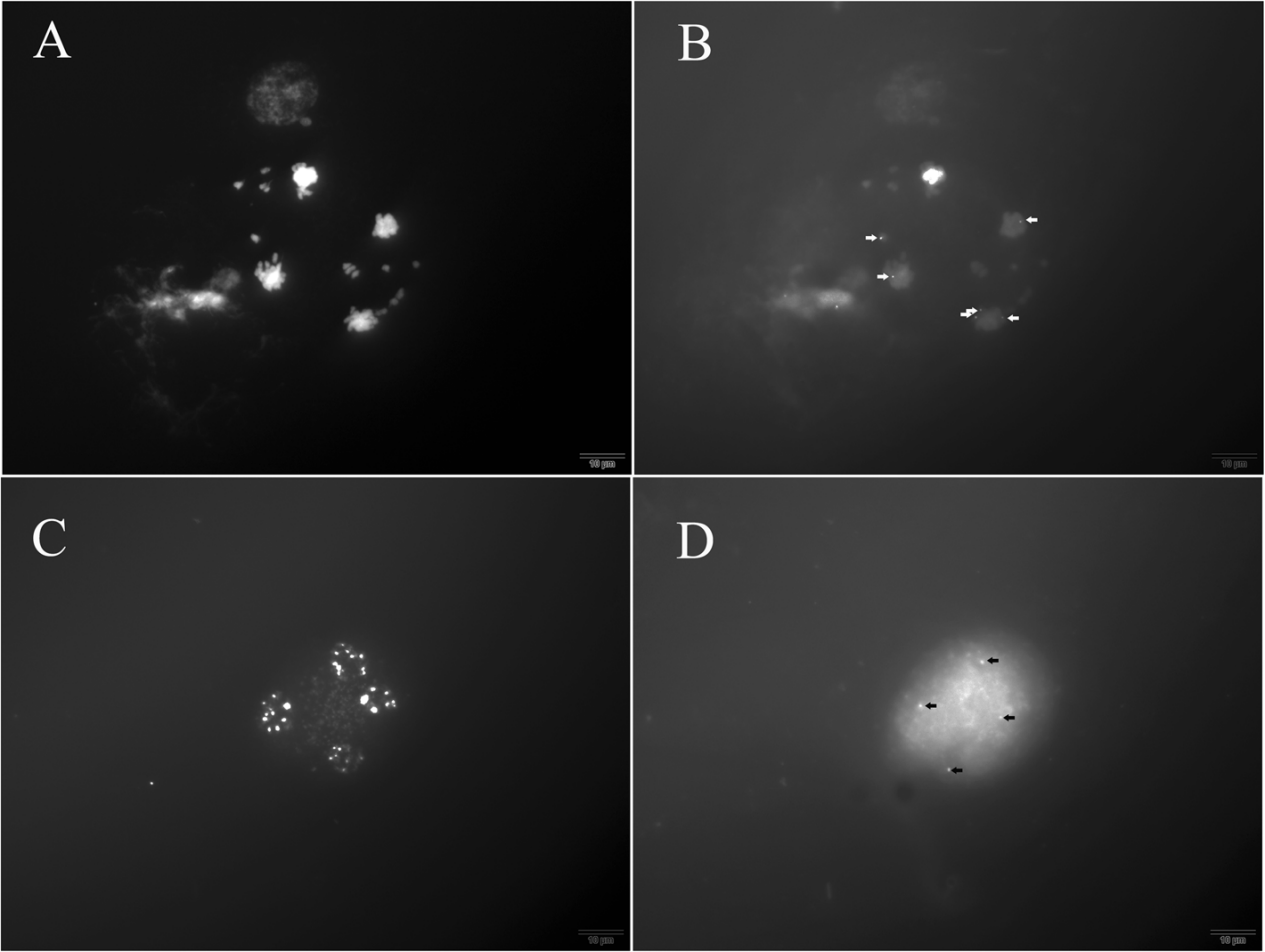
Location of the 5 s rDNA conserved sequence in anaphase II chromosomes of the *Nicotiana* hybrid (**a**, **b**) and in tetrad stage chromosomes of diploid cabbage (**c**, **d**)

A 5 s rDNA conserved sequence was successfully located on meiotic spreads from the *Nicotiana* hybrid, cabbage and maize using FISH (Figs. 4 and 5, arrows). Obvious signals were detected in cabbage and *Nicotiana* hybrid spreads (Fig. 4b, f; Fig. 5b, d). Signals were detected on maize spreads even with the interference from the thick cytoplasm (Fig. 4d). In our experiments, 50–200 satisfactory spreads with obvious signals were obtained on every treated slide. The most satisfactory spreads occurred on the *Nicotiana* hybrid slides. This might be related to the weak interference from the thin cytoplasm.

### Identification of the *Nicotiana plumbaginifolia* genome in the *Nicotiana* hybrid

**Fig. 6:**
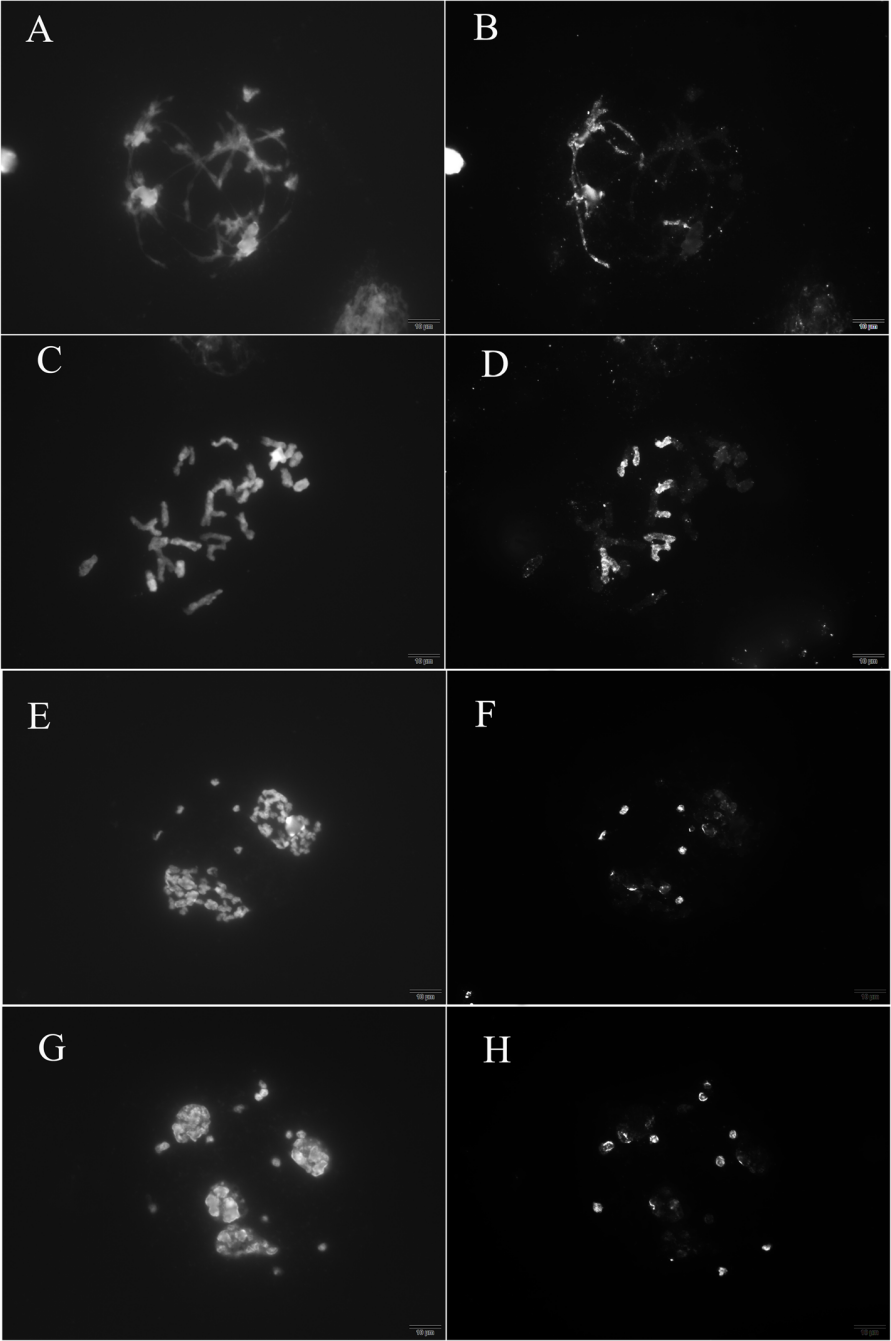
*N. plumbaginifolia* genome identification in *Nicotiana* hybrid meiotic spreads. **a**, **b** Pachytene stage; **c**, **d** Diakinesis/metaphase; **e**, **f** Telophase I; **g**, **h** Tetrad stage; **a**, **c**, **e**, **g** DAPI staining; **b**, **d**, **f**, **h** Signals from probes

Signals from the *N. plumbaginifolia* genome were detected on almost every spread from the *Nicotiana* hybrid. More than 80 satisfactory spreads were obtained on every treated slide. As a result, GISH figures from the prophase to tetrad stage of the *Nicotiana* hybrid were obtained and the *N. plumbaginifolia* genome was easily distinguished by GISH at the different stages (Fig. 6).

## Discussion

### Uniform wall elimination in suspension

Protoplast preparation is widely used [22] and it was employed using our method. PMCs are loosely connected to each other in the anther [23]. Thus, they are easily squeezed from anthers, and they are easily dispersed in suspension as single cells using a light shock. Lower levels of PMC accumulation occurred when PMCs were incubated in a mixed enzyme solution in bottles with flat bottoms. Shaking at times optimized the method. In this manner every PMC’s wall could be digested completely.

### Application prospects for this modified method

Our method was successfully used to prepare meiotic chromosomes from a *Nicotiana* plant, cabbage, and maize. *Nicotiana* plants have been usually used for allotetraplod evolutionary research and classification studies based on cytogenetic analyses [24–26]. Cabbage is a plant in *Brassica*, which is very important for vegetable and oil production. *Brassica* plants are widely used in cytogenetic research [27–29]. Maize is also a model plant for plant cytogenetic research [30–32]. Meiosis is one topic of cytogenetic research. Thus, this modified method would contribute to the cytogenetic analyses of these widely studied plants.

### Possible applications in large-scale cytogenetic analyses

Currently, the heterogeneous PMCs and their daughter cells in many plants, such as some polyploid plants and some interspecific hybrids, have become objects of cytogenetic research [18, 33]. Accurate results should be obtained from the analysis of massive numbers of spreads instead of relying on one or a few cells. Although the existing methods can easily analyze homogeneous cells and are sufficient for simple analyses of heterogeneous cells, they are difficult to apply to the molecular cytogenetic analysis of heterogeneous cells because of PMC wall interference. The interference limits the accumulation of enough satisfactory spreads. Through our modified method, PMC walls were completely and uniformly eliminated, and the spread density can be adjusted. These spreads were successfully used in FISH and GISH processes. This makes it possible to collect massive amounts of information from a few slides. Thus, the modified method could be used in large-scale cytogenetic analyses of heterogeneous PMCs and their daughter cells.

### An easily mastered method

There were no technical problems in the modified process. First, PMCs can be easily squeezed from anther. The following steps require accessible materials, such as flat-based bottles, water bath, centrifuge, and alcohol lamp. Only the mouth blowing at the spreading step requires some technical expertise. In our experience, this method can be easily mastered. Even beginners from two of our collaborator’s laboratories obtained nice spreads on the first attempt.

### Disadvantages of the modified method

There are still some disadvantages to this modified method. First, it is easier to squeeze PMCs from larger anthers with more PMCs, such as tobacco, than from smaller anthers with fewer PMCs, such as cabbage. Thus, we advise that more anthers could be needed if the anther is small and contains only a few PMCs. Anther wall tissue can remain in the PMC suspension, because somatic cells and PMCs can be easily distinguished. Second, some PMCs with separated protoplasts, such as maize PMCs after telophase II, are not suited to be treated by this method because the integrity of the PMCs will be destroyed when the wall is eliminated. A less severe digestion process may be used to avoid this disadvantage. Third, the chromosome spreading at the last step requires technique. To make the results from different operators more uniform, an instrument, such as an airbrush, may be used as an assistive device. However, the production of a suitable instrument is time consuming. We will test these in future work. Fourth, there were 800–1900 spreads on every slide, but only 50–200 satisfactory FISH spreads were obtained. Thus, the treatments before hybridization need to be improved to obtain more satisfactory FISH images on a slide.

## Conclusion

The modified method can eliminate PMC walls completely and uniformly. It produced meiotic chromosome preparations from three widely and well-researched plants. Using this method, massive spreads without PMC wall interference were obtained on one slide. Spreads of all three plants prepared through this method were suitable for the location of a 5 s rDNA conserved sequence by FISH. The *Nicotiana* hybrid spreads were also suitable for the identification of a parental genome by GISH.

## Methods

### Plant materials

A *Nicotiana* hybrid (2n = 58), diploid (2n = 2× = 18), and tetraploid (2n = 4× = 36) cabbage (*Brassica oleracea* L.), and Maize (*Zea mays* L., 2n = 2× = 10) were used as materials. The *Nicotiana* hybrid, which was obtained from the hybridization of *N. tabacum* Lin. octoploid (2n = 8× = 96) and *N. plumbaginifolia* (2n = 2× = 20), was created in our laboratory. Diploid and tetraploid cabbage plants were from the field of the College of Horticulture and Landscape in Southwest University. Maize was from farmland near Southwest University.

### Spread preparation

Slide preparation was performed mainly according to Nagata and Takebe [34], Liang and Li [35], Zhong *et al*. [11], Yang [12], and Kirov *et al*. [36].

1. Flower buds undergoing meiosis were collected at 7:00–9:00, fixed in Carnoy’s fluid [methyl alcohol:acetic acid (V:V = 3:1)] overnight, and stored in 4 °C until the following step.

2. To clear away the Carnoy’s fluid, anthers were washed in distilled water twice after soaking in distilled water for 20 min, cut into two sections, and dipped into 200 μL distilled water in polyethylene centrifuge tubes. Tweezers were used to squeeze PMCs into the distilled water. Then, tubes were lightly shaken on a shaker. Anther walls and other fragments were removed using tweezers and the PMC suspension was obtained. Five *Nicotiana* hybrid anthers, four maize anthers, and six cabbage anthers were used once.

3. The PMC suspension was centrifuged at 2,000 × *g* for 3 min, and the supernatant was removed using a pipettor. The precipitate was resuspended in 300 μL mixed enzyme solution (3 % cellulose + 0.3 % pectinase + 1 % snailase, W/V). The suspension was transfer into 5-cm-high tiny cylindrical bottles with flat bottoms and a 0.8-mm radius. Bottles were capped and vertically immersed in a 37 °C water bath for 1.5 h (maize), 2 h (cabbage), or 2.5 h (*Nicotiana* hybrid), and slightly shaken at times.

4. The suspension was transferred into polyethylene centrifuge tubes and centrifuged at 2000 × *g* for 3 min. The supernatant was removed and the precipitate was resuspended in 200 μL distilled water. Centrifugation was performed again as in the previous step. The precipitate was resuspended in 100 μL Carnoy’s fluid. Then, 10 min later, 10 μL of suspension was dropped on every greaseless slide, immediately dispersed for a short time by forcefully blow from mouth, and dried rapidly using an alcohol flame. Slides were stained with 5 % Giemsa stain. In total, nine to ten slides were prepared at a time.

### Location of the 5 s rDNA conserved sequence by FISH

FISH was performed according to Brammer *et al*. [37] with some modifications. Slides were treated with 20 μg/mL pepsin for 8 min (cabbage), 10 min (*Nicotiana* hybrid), or 50 min (maize) at room temperature. A 20-bp 5 s rDNA conserved sequence (Patent number: CN103409523A) was used as probe. The probe was labeled with 6-carboxytetramethylrhodamine at the 5′-end (Sangon Biotech, Shanghai, China). The probe was dissolved in 2× saline sodium citrate (SSC) as a hybridization solution at a final concentration of 10 ng/μL. The hybridization solution was added to spreads and they were incubated at 37 °C for 4 h. Slides were rinsed in 2× SSC three times for 3 min each time, in 4× SSC (+0.2 % Twain 20 V/V) once for 3 min, and then once in sterile distilled water for 1 min. After air drying, the probe was directly detected under a fluorescent microscope (Olympus, Japan). Photos were taken using a charge-coupled device camera.

### Identification of the *N. plumbaginifolia* genome in hybrid meiotic spreads by GISH

GISH was carried out according to Brammer *et al*. [37] with some modifications. Slides were treated with 20 μg/mL pepsin for 10 min at room temperature. Genomic DNA of *N. plumbaginifolia* was used as the probe, and *N. tabacum* cv. Yunyan87’s DNA was the blocking DNA. Probes were labeled by random priming using Fluorescein-High Prime (Roche, Mannheim, Germany). The final concentration of the probe in the hybridization mixture was 0.25 ng/μL, and the concentration of the blocking DNA was 2.5 ng/μL. The probe was directly detected under a fluorescent microscope (Olympus). Photos were taken using a charge-coupled device camera.

## Abbreviations

GISH: Genomic *in situ* hybridization
FISH: Fluorescence *in situ* hybridization
ISH: *In situ* hybridization
PMC: Pollen mother cell
SSC: Saline sodium citrate
